# MiR-574-5p promotes cell proliferation by negatively regulating small C-terminal domain phosphatase 1 in esophageal squamous cell carcinoma

**DOI:** 10.22038/IJBMS.2022.65886.14492

**Published:** 2022-10

**Authors:** Chunming Zhao, Jialin Liu, Yong Xu, Jiamei Guo, Liping Wang, Linfeng Chen, Lina Xu, Guokai Dong, Wei Zheng, Zhouru Li, Hongxing Cai, Shanshan Li

**Affiliations:** 1 Department of Human Anatomy, Xuzhou Medical University, Xuzhou, Jiangsu, China; 2 Jiangsu Medical Engineering Research Center of Gene Detection, Xuzhou Medical University, Xuzhou, Jiangsu, China; 3 Department of Forensic Medicine, Xuzhou Medical University, Xuzhou, Jiangsu, China; 4 Department of Basic Pathology, Pathology College, Qiqihar Medical University, Qiqihar, Heilongjiang, China; 5 NGS Center, Hangzhou D.A. Medical Laboratory Co., Ltd., Hangzhou, Zhejiang, China; 6 Department of Orthopedics, the Affiliated Hospital of Xuzhou Medical University, Xuzhou, China

**Keywords:** Cell proliferation, CTDSP1 protein, Esophageal squamous cell - carcinoma, MiR-574-5p, Oncogene protein v-akt

## Abstract

**Objective(s):**

Esophageal cancer is one of the most common cancers with high incidence and mortality rates, especially in China. MicroRNA (miRNA) can be used as a prognostic marker for various human cancers. This study aims to detect suitable miRNA markers for esophageal squamous cell carcinoma (ESCC).

**Materials and Methods:**

Our previous gene expression data of ESCC cells and the data from GSE43732 and GSE112840 were analyzed. The expression of miR-574-5p in ESCC patients and controls was analyzed by real-time quantitative PCR. The effect of miR-574-5p on proliferation was detected by real-time cell analysis (RTCA) and EdU proliferation assay after cell transfections. The target gene small C-terminal domain phosphatase 1 (CTDSP1) of miR-574-5p was validated by luciferase reporter assay and western blotting.

**Results:**

In the current study, the bioinformatics analysis found miR-574-5p up-regulated in ESCC. The qPCR assay of 26 ESCC and 13 adjacent/ normal tissues confirmed these results. We further demonstrated that miR-574-5p overexpression promoted cell proliferation. Then the dual-luciferase reporter assay and the rescue experiment suggested that CTDSP1 was a direct target of miR-574-5p.

**Conclusion:**

MiR-574-5p played an oncological role in ESCC by interacting and negatively regulating CTDSP1. These results provided a deeper understanding of the effect of miR-574-5p on ESCC.

## Introduction

Esophageal cancer is the eighth most common cancer and the sixth leading cause of cancer death in the world. Esophageal squamous cell carcinoma (ESCC) accounts for about 87% of all cases of esophageal cancer (1-2). The poor survival rate of ESCC, on one hand, is due to the malignancy of tumor cells; on the other hand, due to the difficulty of early diagnosis. Most of ESCCs are already in the middle or late stage when diagnosed, characterized by deep infiltration and metastasis. Therefore, finding the prognostic markers for ESCC is an important target. 

MicroRNA (miRNA) can be used as a prognostic marker for human cancers. They are relatively stable in abnormal temperature and acid-base environments and are conveniently testable in tissue and blood samples of cancer patients. It is reported that decreased expression of miRNA-187 can be a diagnostic marker for metastatic prostate cancer (3). Down-regulation of serum Let-7, miR-29a, and miR-335 could be promising biomarkers for Breast cancer (4-5). The conjoint analysis of circulating miR-221 and alpha-fetoprotein performs (AFP) increased the accuracy of hepatocellular carcinoma diagnosis (6).

In the current study, we reviewed the previous gene expression data of ESCC cell TE-1 and normal esophageal cell HET-1A and found miR-574-5p up-regulated in ESCC. Multiple articles reported the function of miR-574-5p. For instance, miR-574-5p promotes the differentiation of human cardiac fibroblasts by targeting ARID3A (7) and attenuates acute respiratory distress syndrome by targeting high-mobility group protein B1(8). Exosomes containing miR-574-5p contribute to liver fibrosis by activating hepatic stellate cells, they promote the expression of α-SMA and COL1A1 mRNA and protein in LX-2 (9). Extracellular miR-574-5p was reported to bind toll-like receptor (TLR) 7/8 in both rheumatoid arthritis (inducing osteoclast differentiation) and lung cancer (regulating PGE2-biosynthesis) (10, 11). MiR-574-5p mediates epithelial-mesenchymal transition (EMT) by direct binding and interacting with vimentin in small cell lung cancer (12). Reports of lung cancer also showed that miR-574-5p acts as an RNA decoy to CUG RNA-binding protein 1 by microsomal prostaglandin E synthase-1 induction (13). It also plays an oncogene role in other tumors, miR-574-5p promotes cell proliferation, migration, and invasion by targeting forkhead box N3 and regulating the β-catenin pathways in both nasopharyngeal carcinoma (14) and thyroid cancer cells (15). It also promotes angiogenesis by targeting protein tyrosine phosphatase non-receptor type 3 in gastric cancer cells (16) and participates in apoptosis, proliferation, cell cycle, etc., by targeting RNA binding protein QKI in both cervical cancer and thyroid cancer cells (17-18). It is reported that miR-574-5p promotes proliferation and inhibits apoptosis of ESCC cells (19). Therefore, miR-574-5p may be a biomarker for ESCC, and the following mechanisms were explored in this study.

The function of most miRNAs is to regulate the post-transcription of messenger RNA (mRNA) through degrading mRNA or inhibiting protein translation. One miRNA may target a variety of mRNAs, while a single mRNA may be targeted by multiple miRNAs, composing the miRNA-mRNA regulatory network. It is reported that miR-634 enhances the radiotherapy sensitivity of breast cancer cells by negatively regulating signal transducer and activator of transcription 3 (STAT3) (20). The long noncoding RNA MEG3 interacts with miR-494 to regulate the gene of phosphate and tension homology deleted on chromosome ten (PTEN) and then suppresses bladder cancer progression (21). MiRNA27a mimics promote cell growth and suppress apoptosis of human pancreatic cancer cells through the Wnt/β-catenin pathway (22). 

In this study, bioinformatics analysis was performed and found small C-terminal domain phosphatase 1 (CTDSP1) as a promising target gene of miR-574-5p. The C-terminal domain (CTD) is the largest subunit of RNA polymerase II (RNAP II), the crucial component of a transcription apparatus, and regulates mRNA modification. The leading function of CTDSP1 is dephosphorylation Ser5 in consensus peptide repeats of RNAP II CTD (23). CTDSP1, along with the other CTD phosphatase (SCP) subfamily members: SCP, CTDSP2, and CTDSPL are involved in many vital cellular processes and tumorigenesis (24). Thus, we hypothesized that miR-574-5p played a crucial role in ESCC progression by regulating CTDSP1. To test this hypothesis, we examined the regulation of miR-574-5p on CTDSP1 in ESCC cell lines.

## Materials and Methods

***Data analysis***

The previous miRNA and mRNA expression data of TE-1 and normal esophageal cell HET-1A (Agilent microarray) were reviewed, and the differentially expressed genes were shown by Heatmap using R software v3.6.3 and package ComplexHeatmap (25). The GSE43732 (26), GSE112840 (27), and GSE23400 (28) data were downloaded from GEO public database (http://www.ncbi.nlm.nih.gov/geo/) and analyzed by the GEO2R tool using the default method. The predicted target genes were queried from Targetscan (http://www.targetscan.org/) (29) under the settings of “Enter a microRNA name”=miR-574-5p, “Select a species”=Human, and “the others”=default and downloaded the results.

Volcano plots were used to show differences, and Venn analysis was performed to get overlapping miRNA or mRNA, respectively. GraphPad Prism 7, R software v3.6.3 and package ggplot2 were used for statistical analysis and visualization. 

***Human tissues and cell lines***

26 ESCC and 13 adjacent/ normal tissues were obtained from the Affiliated Hospital of Xuzhou Medical University, China. The tissues were stored at -80 °C after surgical removal. The relevant clinical data were collected by retrospective review of patient files. The experiment was approved by the Ethics Committee of the Affiliated Hospital of Xuzhou Medical University (XYFY2022-KL103). The human ESCC cell lines TE-1 and ECA-109 (from Public Experimental Research Center, Xuzhou Medical University, China) were cultured in 1640 medium (10% fetal bovine serum and 100 U/ml of penicillin-streptomycin). 293T cells (from the Department of Hematology, The Affiliated Hospital of Xuzhou Medical University, China) were cultured in DMEM medium (10% fetal bovine serum and 100 U/ml of penicillin-streptomycin).

***Cell transfections***

The has-miR-574-5p mimics (mimics, Sense UGAGUGUGUGUGUGUGAGUGUGU, Antisense ACACUCACACACACACACUCAUU) and the negative control (NC, Sense UUGUACUACACAAAAGUACUG, Antisense GUACUUUUGUGUAGUACAAUU) were synthesized by Sangon Biotech, China. The wild-type (WT) 3′-UTR and mutation-type (MUT) 3′-UTR of CTDSP1 were cloned into the psiCHECK2 luciferase reporter vector. CTDSP1 mRNA was cloned into the pcDNA3.1(+) vector (Hunan Fenghui Biotechnology Co., Ltd, Hunan, China). 1×10^5^ cells were seeded in a six-well plate before transfection. Transfections were performed with Lipofectamine 2000 (Thermo Fisher Scientific, Inc., USA) according to the manufacturer’s protocol when the cell density reached 30%–50%. The following experiments were performed 48 hr later.

***RNA extraction and reverse transcription-quantitative PCR (qPCR)***

MiRNA from ESCC tissues and cells were extracted with miRcute miRNA Isolation Kit (Tiangen Biotech, Beijing, China) according to the manufacturer’s protocol. The miRNA (2 μg) was reverse transcribed with a miRcute Plus miRNA First-Strand cDNA Kit (poly(dA) tailing method, Tiangen Biotech, Beijing, China). The obtained first-strand cDNA was then amplified by the ABI 7500 Real-Time PCR system (Applied Biosystems; Thermo Fisher Scientific, Inc.) with a miRcute Plus miRNA qPCR Kit (Tiangen Biotech, Beijing, China). The primers of hsa-miR-574-5p were from Tiangen Biotech (Beijing, China). DdH_2_O was used as a negative control. The results were normalized against the expression level of U6 (Tiangen Biotech, Beijing, China).

Total RNA from the ESCC cells was extracted with Trizol reagent (Tiangen Biotech, Beijing, China) according to the manufacturer’s instructions. The RNA (2 μg) was reverse transcribed with a cDNA Reverse Transcription kit (Tiangen Biotech, Beijing, China). The obtained cDNA was then amplified with the ABI 7500 Real-Time PCR system (Applied Biosystems; Thermo Fisher Scientific, Inc.) by a Universal SYBR Green Mix (ABclonal, Wuhan, China). DdH_2_O was used as a negative control. The results were normalized against the expression level of Actb (Sangon Biotech, Shanghai, China). 

All data were measured by the comparative quantification (ΔΔCt) method. The qPCR reactions were performed in triplicate, and three independent RNA samples from cells were assayed. The primers were as follows: MAP1LC3B F: TACTGTCAAAGCCATCCTGATT, R: CGATGGGTCTT

GTTGAAAATGT; CTDSP1 F: ACCTGCCTCCTATGTCTT

CCATCC, R: CACTGAGTACACGTCGTCCACAC; PRKDC

F: TGGCACGAGGAAGCAAAGATCAC, R: GGTCCATCAGG

CACTTCACTTGAG; COBLL1 F: CCCAAGACTTTGC ACACATCCAG, R: TGAACCTGCCCTCACTCTTCCTG.

***Protein preparation and immunoblot analysis***

The cells were collected and suspended in cell lysis buffer (Beyotime Institute of Biotechnology, Shanghai, China) on a rotator of 4 °C for 30 min. The cells were then homogenized and centrifuged at 12,000 rpm, 4 °C for 15 min. The resulting supernatant was collected. The protein concentration was determined with a BCA Protein Assay kit (Beyotime Institute of Biotechnology, Shanghai, China) according to the manufacturer’s protocol.

20 μg of total protein was separated by 10% sodium dodecyl sulfate-polyacrylamide gel electrophoresis, then transferred to a polyvinylidene difluoride (PVDF) membrane (Merck Millipore Ltd, Darmstadt, Germany). After blocking with 5% non-fat milk, the PVDF membrane was incubated with primary antibody overnight at 4 °C. The specifications of antibodies used in western blot were as follows: mouse anti-AKT antibody, rabbit anti-P AKT (ser 473) antibody, rabbit anti-CTDSP1 antibody (Proteintech Group, Wuhan, China, 1:1000); the secondary antibody goat anti-rabbit and goat anti-mouse antibody (Jackson ImmumoResearch, PA, US,1:10000). Mouse anti-β actin antibody (Proteintech Group, Wuhan, China, 1:5000) was used as a loading control.

***Real-time cell analysis (RTCA)***

Survival assay was performed by the xCELLigence RTCA system (ACEA Biosciences, USA) according to the manufacturer’s protocol. Specifically, 5×10^3 ^cells were seeded in a well of E-16-plate. The plate was then connected to the RTCA system in an incubator. After 30 min pre-culture, the Cell Index was recorded every 15 min until 72 hr. The assays were performed in triplicate, and three independent samples from cells were assayed.

***EdU proliferation assay***

EdU proliferation assay was detected with Click-iT™ EdU Alexa Fluor™ 488 Flow Cytometry Assay Kit (ThermoFisher) according to the manufacturer’s instructions. Specifically, after 2 hr of 10 μM EdU culture, cells were fixed and permeabilized using a BD transcription factor buffer set. And then incubated with click-iT working solution light-protected for 30 min at room temperature. A BD LSR Fortessa flow cytometer was used for flow cytometry (BD Biosciences). FACS data were analyzed by FlowJo software (Tree Star).

***Dual-luciferase reporter assay***

The consequential pairing of the target region of has-miR-574-5p and CTDSP1 was predicted by Targetscan (http://www.targetscan.org/)^(^29^)^. The WT 3′-UTR and MUT 3′-UTR of CTDSP1 were cloned into the psiCHECK2 luciferase reporter vector (Hunan Fenghui Biotechnology Co., Ltd, Hunan, China). 293T Cells were cultured in 96-well plates (5×10^4^ cells/well) and co-transfected with miR-574-5p mimics/ NC and luciferase reporter with WT or MUT CTDSP1 3′- UTR with Lipofectamine 2000 (Thermo Fisher Scientific, Inc., USA). RLU (Relative luciferase activity) was determined by the ratio of Firefly luciferase to Renilla luciferase with the dual-luciferase reporter assay kit (Beyotime, China) according to the manufacturer’s instructions. 

***Statistical analysis***

SPSS for Windows 24.0 was used for all statistical tests and calculations. The data were reported as mean±SD. Differences were assessed using one-way ANOVA analysis followed by the t-test, if it was normal distribution or the Mann-Whitney U test if it was non-normal distribution. Values of *P*<0.05 were considered statistically significant. All statistical graphs were exhibited by GraphPad Prism 7.

## Results

***MiR-574-5p was highly expressed in ESCC***

**Figure 1:**
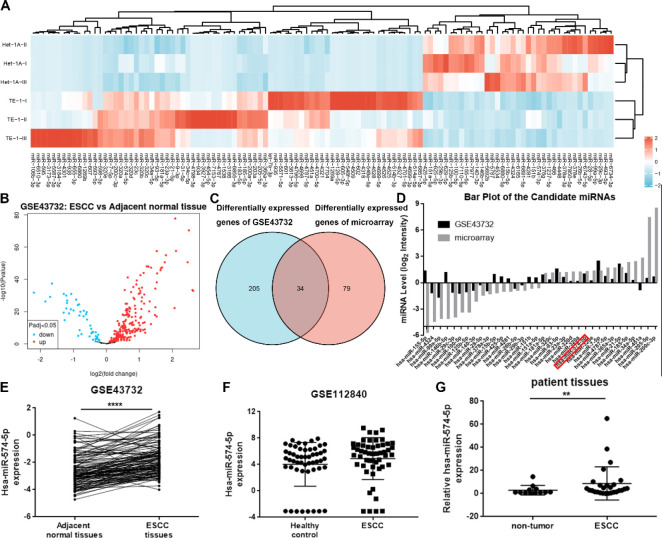
MiR-574-5p was highly expressed in ESCC. A. Heatmap of the differentially expressed miRNAs between TE-1 and normal esophageal cell HET-1A. B. Volcano plots show the differentially expressed miRNAs between ESCC and adjacent normal tissues from ESCC patients (GSE43732 dataset). C. Venn diagram shows 34 overlaps of differentially expressed miRNAs of our microarray and GSE43732 data. D. Expressions of overlapping miRNAs of our microarray and GSE43732 data. E. MiR-574-5p expression in ESCC tissues and the adjacent paired tissues (GSE43732 dataset, ****P<0.0001). F. MiR-574-5p was highly expressed in the serum of patients with ESCC according to GSE112840 data. G. qPCR assay showed miR-574-5p level of 26 ESCC and 13 adjacent/normal tissues (mean±SD, **P<0.01)

We have reported gene expression data of TE-1 and normal esophageal cell HET-1A and found 113 miRNAs of TE-1 cells significantly different from those of HET-1A cells, including 76 up-regulated and 37 down-regulated miRNAs (Figure 1A). Volcano plots showed the differentially expressed miRNAs of the GSE43732 (26) dataset by the GEO2R tool, which assesses the miRNA expression profiles of 119 ESCC patients (Figure 1B). The Venn diagram in Figure 1C shows 34 overlaps of differentially expressed miRNAs from microarray and GSE43732, and Figure 1D displays the miRNAs. We further analyzed miR-574-5p expression data from the GSE43732 dataset and found that miR-574-5p was highly expressed in ESCC tissues compared with the adjacent paired tissues (Figure 1E). The clinical data from the GSE43732 dataset (119 patients) were then analyzed and the baseline data were shown in Table 1. Interestingly, differences between the miR-574-5p high and low expression groups were not statistically significant, except for the tumor location. MiR-574-5p was continuously validated in the GSE112840 dataset, which showed the miRNAs expression profiles in the serum of ESCC patients. MiR-574-5p was one of the up-regulated miRNAs and was recognized as a serum biomarker of ESCC (Figure 1F) (27). Therefore, the miR-574-5p level was detected in 26 ESCC and 13 adjacent/ normal tissues by using qPCR assay. The results showed that the expression of miR-574-5p was significantly overexpressed in ESCC tissues compared with non-tumor tissues (Figure 1G). 

***MiR-574-5p promoted cell proliferation***

**Figure 2:**
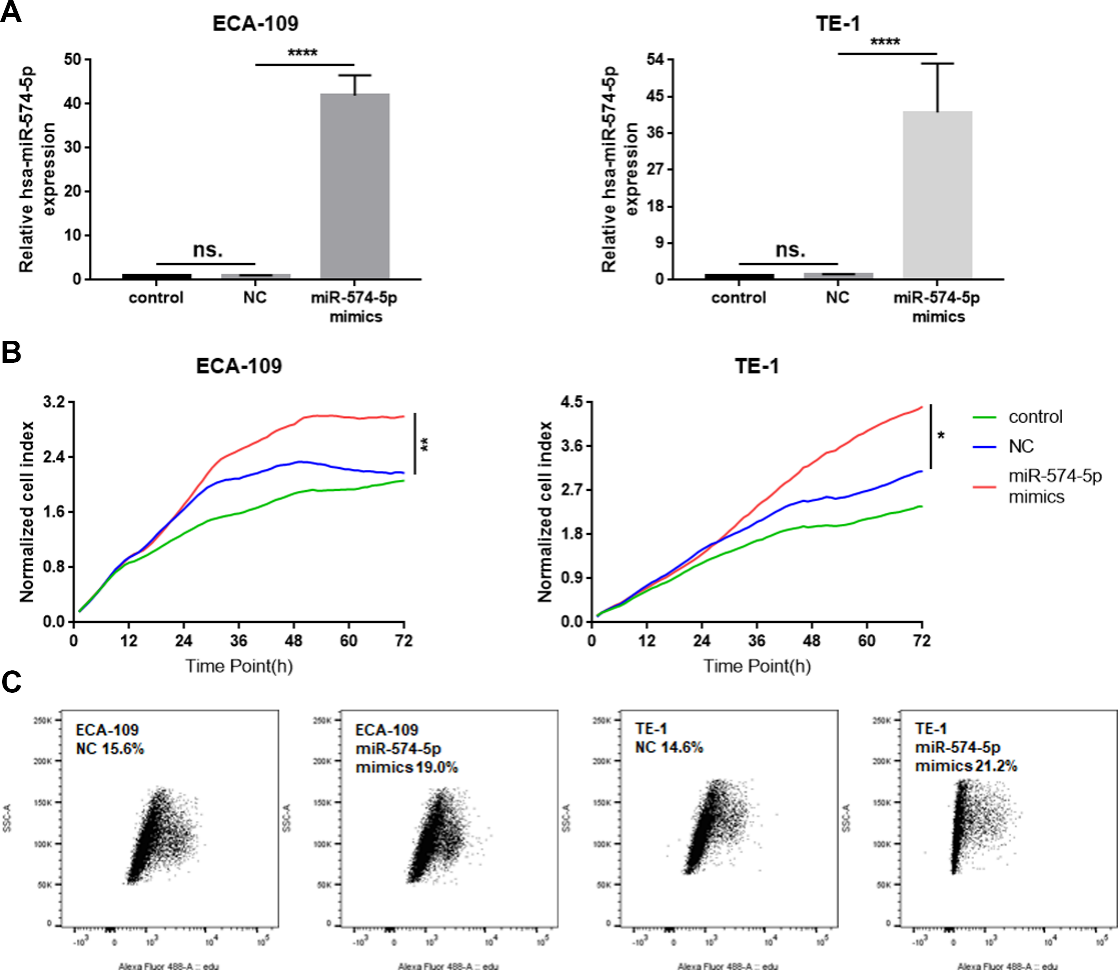
MiR-574-5p promoted cell proliferation. A. qPCR assay confirmed the high expression of miR-574-5p after miR-574-5p mimics transfection in both TE-1 and ECA-109 cell lines (mean±SD, ****P<0.0001). B. RTCA showed the survival curves of TE-1 and ECA-109 cells after miR-574-5p mimics transfection (*P<0.05, **P<0.01). C. Proliferating cells were labeled with EdU and quantified by flow cytometry after miR-574-5p mimics transfection

To detect the effect of miR-574-5p on ESCC cells, the miR-574-5p mimics and negative control (NC) were synthesized and transfected into TE-1 and ECA-109 cell lines. qPCR assay was performed and showed miR-574-5p significantly overexpressed after transfection (Figure 2A). The survival assay was tested by RTCA. RTCA measures the changes in electrical impedance of adherent monolayer cells using cell culture microplates containing microelectrodes. It provides dynamic real-time, label-free, and non-invasive analysis of cellular events such as cell adhesion, spreading, proliferation, cell viability, cell death, and detachment. The cell index value (CI) is used to record the electrical impedance of cells^(^30^)^. In this study, the CIs were recorded every 15 min until 72 hr, and the curves of normalized CIs were performed. The results showed that miR-574-5p mimics significantly increased the CIs compared with the control or NC group (Figure 2B). Cell proliferation was detected by EdU proliferation assay. Flow cytometry results showed that the EdU^-^positive cells were increased in the miR-574-5p mimics group (Figure 2C). Thus, we believed that miR-574-5p promoted the proliferation of TE-1 and ECA-109 cells.

***CTDSP1 was a direct target of miR-574-5p***

**Figure 3:**
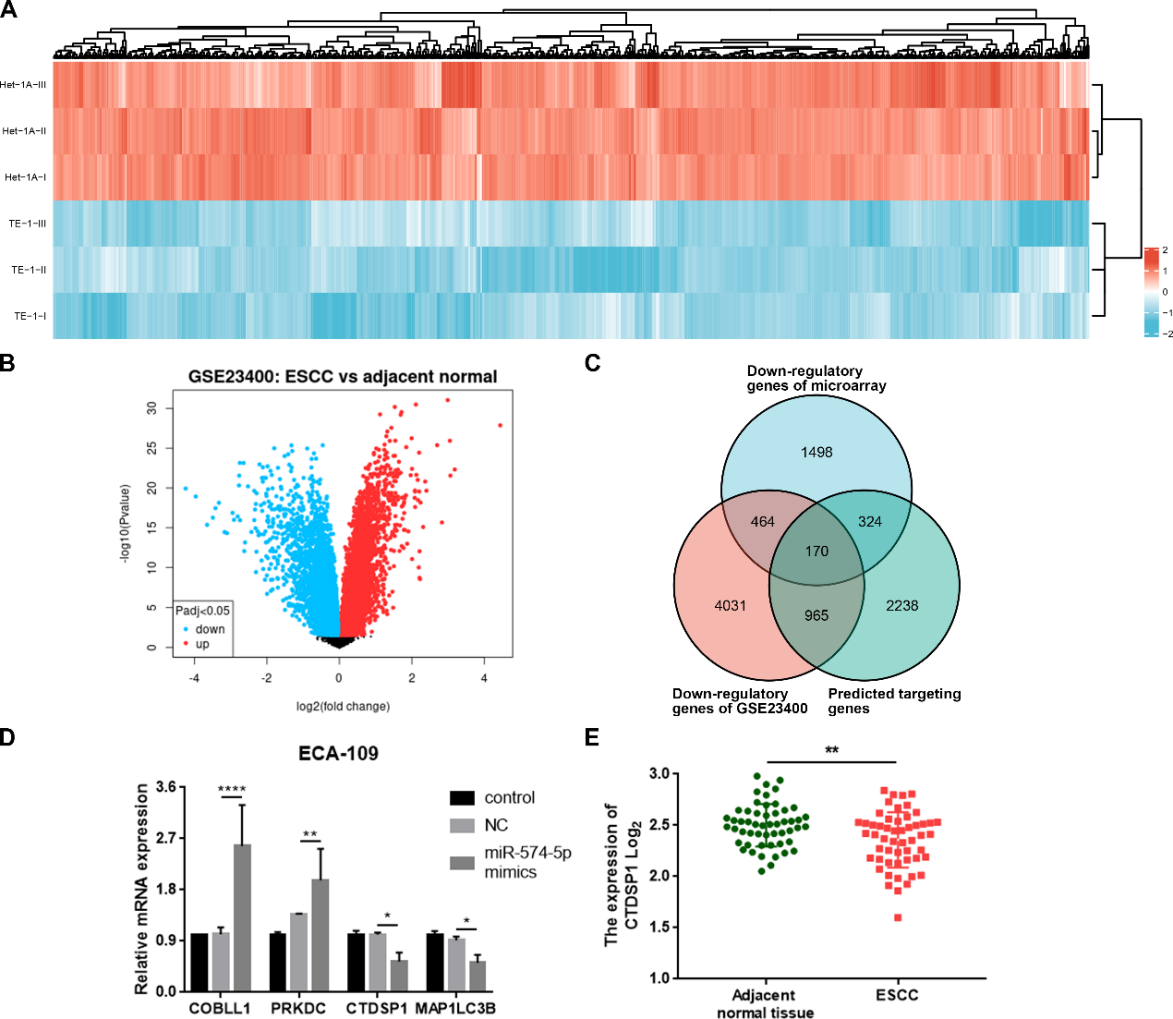
CTDSP1 was a target of miR-574-5p. A. Heatmap of the differentially expressed genes between TE-1 and normal esophageal cell HET-1A. B. Volcano plots showed the differentially expressed genes between 53 ESCC and 53 matched normal samples from ESCC patients (GSE23400 dataset). C. Venn Diagram showed the overlaps of GSE23400, our microarray data, and the possible target genes from targetscan. D. qPCR assay showed the expressions of a few genes of the overlaps after miR-574-5p mimics transfection (mean±SD, *P<0.05, **P<0.01, ****P<0.0001). E. CTDSP1 expression in GSE23400 dataset (**P<0.01)

The down-regulatory genes of the previous microarray were shown by Heatmap (Figure 3A). Volcano plots showed the differentially expressed mRNAs of the GSE23400^(^28^)^ dataset by the GEO2R tool, which analyzed 53 ESCC and 53 matched normal samples (Figure 3B). We looked for the target genes of has-miR-574-5p on Targetscan^(^29^)^ and found 2841 matching results. A Venn Diagram was then made with the targetscan, GSE23400, microarray data, and 170 genes may meet our needs (Figure 3C). After literature research, four genes: Cordon-Bleu WH2 Repeat Protein Like 1 (COBLL1), Protein Kinase DNA-Activated Catalytic Subunit (PRKDC), Microtubule Associated Protein 1 Light Chain 3 Beta (MAP1LC3B), and CTDSP1 were verified by qPCR assay. The results showed that miR-574-5p mimics up-regulated COBLL1 and PRKDC but down-regulated MAP1LC3B and CTDSP1 (Figure 3D). CTDSP1 is a protein phosphatase. It is reported that down-regulation of CTDSP1 may promote cancer cell migration and invasion (23). The result was further confirmed by the differentially expressed data of CTDSP1 in ESCC patients’ tissues from the GSE23400 dataset, which showed that CTDSP1 markedly decreased in ESCC tissues compared with normal esophageal tissues (Figure 3E). 

**Figure 4:**
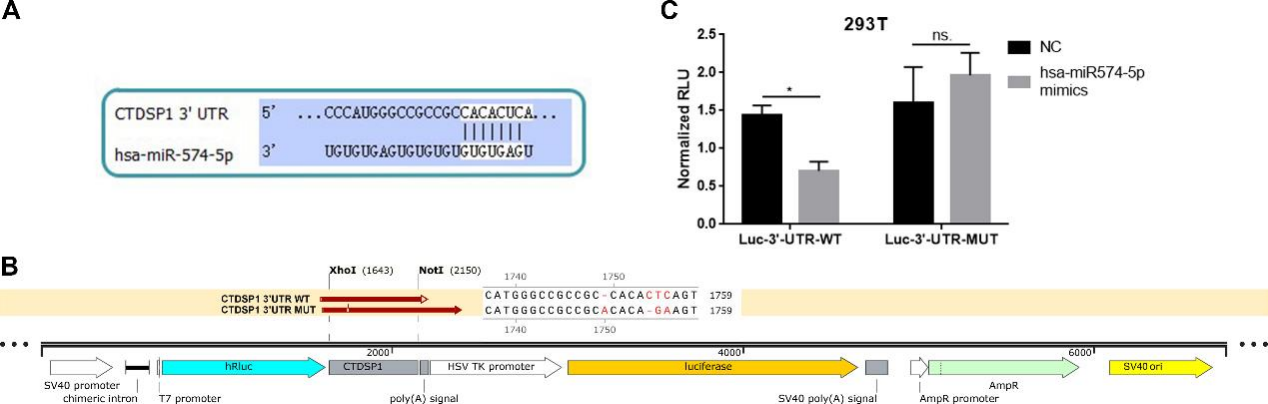
MiR-574-5p directly interacted with CTDSP1. A. Predicted binding site of miR‐574‐5p and CTDSP1 3′-UTR. B. WT and MUT vector of CTDSP1 3′-UTR. C. Dual-luciferase reporter assay showed the RLU of cells transfected with miR‐574‐5p mimics and/or 3′-UTR of CTDSP1 (WT or MUT) (mean±SD, *P<0.05)

We further tested the interaction between miR‐574‐5p and CTDSP1. The predicted binding site of miR‐574‐5p and CTDSP1 3′-UTR was shown in Figure 4A, and the WT and MUT vector of CTDSP1 3′-UTR were designed accordingly (Figure 4B). Dualluciferase reporter assay performed on 293T cell line revealed that the RLU of cells transfected both miR‐574‐5p mimics and vector with WT 3′-UTR of CTDSP1 significantly decreased compared with those of the other three groups (mimics+ MUT, NC+ WT, NC+ MUT) (Figure 4C). The decreased RLU, caused by luciferase gene transcription decrease, indicated the binding of miR‐574‐5p and CTDSP1 3′-UTR. In addition, the RLU remained stable in the groups using the substitution of NC and/ or MUT 3′-UTR, which indicated the binding of miR‐574‐5p and CTDSP1 3′-UTR was specific. These results suggested the direct interaction between miR‐574‐5p and CTDSP1.

***MiR-574-5p promoted cell proliferation by negatively regulating CTDSP1***

**Figure 5:**
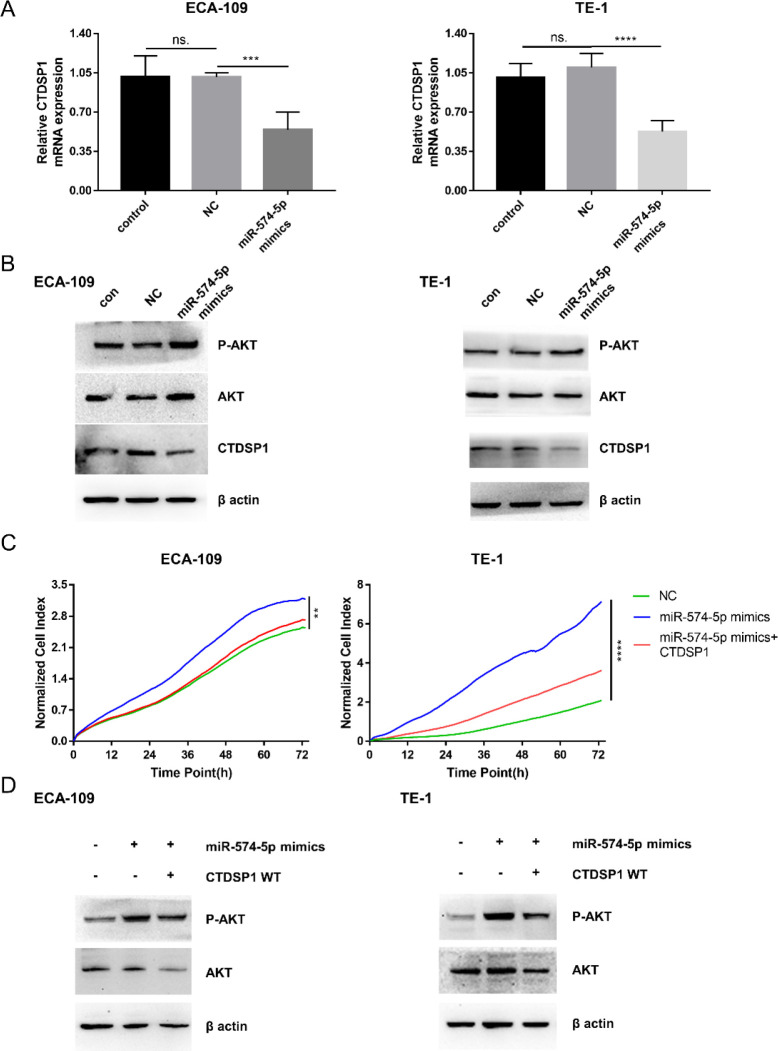
MiR-574-5p promoted cell proliferation by negatively regulating CTDSP1. A. qPCR assay showed the CTDSP1 mRNA expression after miR‐574‐5p mimics transfection (mean±SD, ***P<0.001, ****P<0.0001). B. Immunoblot of CTDSP1, AKT, and phosphorylated AKT (P AKT) after miR‐574‐5p mimics transfection. C. RTCA showed the survival curves of cells transfected with miR‐574‐5p mimics and CTDSP1 (**P<0.01, ****P<0.0001). D. Immunoblot of AKT and P AKT after miR‐574‐5p mimics and CTDSP1 transfection

qPCR and immunoblot were done to confirm the regulatory relationship between miR‐574‐5p and CTDSP1 expression. The mRNA expression of CTDSP1 was markedly decreased in the miR-574-5p mimics group in both TE-1 and ECA-109 cells (Figure 5A). This result was further supported by immunoblot. Moreover, miR-574-5p mimics promoted AKT phosphorylation (Figure 5B), which may be because of the reduction of CTDSP1 (31).

We further detected the necessity of CTDSP1 in the regulation of cell proliferation by miR-574-5p. RTCA assay confirmed that the CIs in the miR-574-5p mimics group increased compared with the NC group, while CTDSP1 counteracted this promoting effect (Figure 5C). Immunoblot results showed that the effect of miR-574-5p on AKT phosphorylation was also reversed by CTDSP1 (Figure 5D). These results suggested that the effect of miR-574-5p on cell proliferation was achieved through CTDSP1.

**Table 1:** Baseline data from the GSE43732 dataset (119 patients)

Characteristic	has-miR-574-5p expression		*p*
	Low	High	
n	60	59	
T stage, n (%)			0.808
1	3 (2.5%)	5 (4.2%)	
2	11 (9.2%)	9 (7.6%)	
3	30 (25.2%)	32 (26.9%)	
4	16 (13.4%)	13 (10.9%)	
N stage, n (%)			0.359
0	23 (19.3%)	31 (26.1%)	
1	25 (21%)	17 (14.3%)	
2	6 (5%)	7 (5.9%)	
3	6 (5%)	4 (3.4%)	
TNM stage, n (%)			0.556
I	3 (2.5%)	3 (2.5%)	
II	21 (17.6%)	26 (21.8%)	
III	36 (30.3%)	30 (25.2%)	
tumor grade, n (%)			0.515
well	14 (11.8%)	9 (7.6%)	
moderately	30 (25.2%)	34 (28.6%)	
poorly	16 (13.4%)	16 (13.4%)	
tumor location, n (%)			**0.003**
upper	13 (10.9%)	1 (0.8%)	
middle	31 (26.1%)	38 (31.9%)	
lower	16 (13.4%)	20 (16.8%)	
tobacco use, n (%)			1.000
yes	40 (33.6%)	40 (33.6%)	
no	20 (16.8%)	19 (16%)	
alcohol use, n (%)			0.653
yes	39 (32.8%)	35 (29.4%)	
no	21 (17.6%)	24 (20.2%)	
family history, n (%)			0.661
positive	12 (10.1%)	9 (7.6%)	
negative	48 (40.3%)	50 (42%)	
gender, n (%)			0.966
male	50 (42%)	48 (40.3%)	
female	10 (8.4%)	11 (9.2%)	
age, mean ± SD	57.8 ± 9.62	60.27 ± 8.06	0.132

## Discussion

In the current study, we found that miR-574-5p played an oncogenic role in ESCC by negatively regulating CTDSP1. According to our previous gene expression data, the miR-574-5p level in TE-1 is significantly higher than in HET-1A (26-27). The following qPCR assays of ESCC tissues and adjacent/ normal tissues confirmed the results. Our findings are consistent with the following reports. A study of serum samples (ESCC patients and age- and sex-matched controls) showed that miR-574-5p is one of the powerful biomarkers for the diagnosis of ESCC (27). Some other studies of tissues (ESCC tissues and adjacent non-tumor tissues) showed similar results (26, 32). These observations motivated us to test the role of miR-574-5p in ESCC.

RTCA and EdU proliferation assays performed on ESCC cells experimentally demonstrate that overexpressed miR-574-5p promoted ESCC cell proliferation *in vitro*. MiR-574-5p is increased both in human ESCC tissues and cell lines, which serves as a tumor promoter: promotes proliferation and inhibits apoptosis by targeting ZNF70 via mitochondrial-mediated ROS generation and MAPK pathways (19). It also participates in competing for endogenous RNA (ceRNA), for instance, lncRNA MTX2-6 inhibited cell proliferation and promoted cell apoptosis of ESCC, which exerts as a ceRNA by binding miR-574-5p and elevates the expression of SMAD4 (33). Our findings are consistent with these reports.

Given that miR-574-5p plays a critical role in regulating cell proliferation, we further investigated the target of miR-574-5p on ESCC cells using bioinformatics analysis and found miR-574-5p has a binding site in the 3′UTR of the CTDSP1 mRNA according to TargetScan prediction. After literature research and qPCR assay, CTDSP1 was finally chosen as a promising target gene for miR-574-5p. CTDSP1 is a nuclear phosphatase mainly located in the nucleus (34), while miR-574-5p has a sequence associated with nuclear localization (35). Moreover, Emmerich, etc., reported that miR-574-5p is located in the nucleus of lung cancer cells (36). These pieces of evidence suggest the possibility of direct interaction between miR-574-5p and CTDSP1, which is further confirmed by the following dual-luciferase reporter assay in this study. 

CTDSP1 is an enzyme that removes a phosphate group from the phosphorylated proteins (34). It participates in various cellular activities such as neuronal gene silencing, cell cycle regulation, and some cell signal transductions (23). A few articles report the molecules that regulate CTDSP1: USP29, one of the deubiquitinases (DUBs) enhances the interaction of Snail and CTDSP1, inducing Snail dephosphorylation and deubiquitination, and preventing Snail degradation in gastric cancer cells (37); 27-hydroxycholesterol (27-HC), the most abundant metabolite of cholesterol, inhibits PP2A and CTDSP1 transcription and then blocks c-Myc Ser62 dephosphorylation in breast cancer cells (38); miR-124 inhibits neuroglioma evolution via down-regulating CTDSP1(39). However, the report on CTDSP1 in ESCC is hard to find. In the current study, CTDSP1 was found decreased in ESCC tissues according to data analysis. Then, both the qPCR assay and the immunoblot showed that miR-574-5p negatively regulated CTDSP1. The RTCA assay showed the counterregulatory effect of CTDSP1 on miR-574-5p promoting proliferation. Thus, the relationship between miR-574-5p and CTDSP1 was established that miR-574-5p promoted proliferation by targeting CTDSP1 in ESCC cells.

We then focused on the downstream regulation of miR-574-5p and CTDSP1. Some articles present the dephosphorylation function of CTDSP1: CTDSP1 regulates the phosphorylation state of Repressor Element 1 Silencing Transcription factor (REST) by preventing the target to the proteasome (40); CTDSP1 interacts with c-Myc both *in vivo *and* in vitro *that dephosphorylates c-Myc Ser62 and negatively regulates cancer cell proliferation (41); CTDSP1 interacts with the N terminus of Twist1, decreases Twist1 Ser68 and total Twist1 proteins, and inhibits the epithelial-to-mesenchymal transition (EMT), the migration and invasion in breast cancer cells (42). 

Here we reported the effect of miR-574-5p and CTDSP1 on AKT phosphorylation. AKT, a serine/threonine kinase, is an intermediate molecule of many vital cell activities, including cell survival, angiogenesis, tumor development, and tumor growth. AKT1, AKT2, and AKT3 are the subtypes of AKT. AKT can be activated through 3-phosphoinositide-dependent protein kinases (PDKs), rapamycin complex 2 (mTORC2), and Integrin-linked kinase (ILK) by phosphorylating Thr308 and Ser473, etc. (43). In this study, miR-574-5p was found to promote AKT phosphorylation, and this promoting effect was reversed by CTDSP1. Our results are consistent with the report that CTDSP1 knockout increases AKT phosphorylation by decreasing the dephosphorylation of AKT Ser473 (31).

## Conclusion

Our study found that miR-574-5p played an oncogenic role in ESCC. MiR-574-5p overexpression promoted cell proliferation by interacting and negatively regulating CTDSP1. These results provided a deeper understanding of the effect of miR-574-5p on ESCC. MiR-574-5p and CTDSP1 can be the prognostic biomarkers and therapeutic targets of ESCC.

## Authors’ Contributions

SL and HC Conceived and designed the work. CZ, JL, YX, JG, GD, and ZL Performed experimental work and collected and analyzed the statistical data. LW, LC, LX, and WZ Collected ESCC tissues and confirmed the pathological diagnosis. CZ and JL Interpreted the results. CZ, SL, and HC Drafted and critically evaluated the manuscript. All authors have read and agreed to the published version of the manuscript.

## Declarations and Ethics Statements

The study was conducted by the Declaration of Helsinki, and approved by the Ethics Committee of the Affiliated Hospital of Xuzhou Medical University (XYFY2022-KL103).

## Availability of Data and Materials

The datasets related to the current study are available from the corresponding author upon reasonable request.

## Conflicts of Interest

The author(s) declared no potential conflicts of interest concerning the research, authorship, and/or publication of this article.
